# Longitudinal associations between blood lysophosphatidylcholines and skeletal muscle mitochondrial function

**DOI:** 10.1007/s11357-022-00548-w

**Published:** 2022-04-07

**Authors:** Qu Tian, Brendan A. Mitchell, Marta Zampino, Luigi Ferrucci

**Affiliations:** 1 Longitudinal Studies Section, Translational Gerontology Branch, National Institute On Aging, Baltimore, MD 21224 USA; 2 251 Bayview Blvd., Suite 100, RM04B316, Baltimore, MD 21224 USA

**Keywords:** Lysophosphatidylcholines, Mitochondrial function, Cardiolipin synthesis

## Abstract

Lysophosphatidylcholines (LPCs) are phospholipids critical in the synthesis of cardiolipin, an essential component of mitochondrial membranes. Lower plasma LPCs have been cross-sectionally associated with lower skeletal muscle mitochondrial function, but whether lower LPCs and their decline over time are longitudinally associated with an accelerated decline of mitochondria function is unknown. We analyzed data from 184 participants in the Baltimore Longitudinal Study of Aging (mean age: 74.5 years, 57% women, 25% black) who had repeated measures of plasma LPCs (16:0, 16:1, 17:0, 18:0, 18:1, 18:2, 20:3, 20:4, 24:0, and 28:1) by liquid chromatography-tandem mass spectrometry and repeated measures of skeletal muscle oxidative capacity (*k*_PCr_) assessed by ^31^P magnetic resonance spectroscopy over an average of 2.4 years. Rates of change in *k*_PCr_ and each LPC were first estimated using simple linear regression. In multivariable linear regression models adjusted for baseline demographics and PCr % depletion, lower baseline LPC 16:1 and faster rates of decline in LPC 16:1 and 18:1 were significantly associated with a faster rate of decline in *k*_PCr_ (*B* =  − 0.169, 95% CI: − 0.328, − 0.010, *p* = 0.038; *B* = 0.209, 95% CI: 0.065, 0.352, *p* = 0.005; *B* = 0.156, 95% CI: 0.011, 0.301, *p* = 0.035, respectively). Rates of change in other LPCs were not significantly associated with change in *k*_PCr_ (all *p* > 0.05). Lower baseline concentrations and faster decline in selected plasma lysophosphatidylcholines over time are associated with faster decline in skeletal muscle mitochondrial function. Strategies to prevent the decline of plasma LPCs at an early stage may slow down mitochondrial function decline and impairment during aging.

**Supplementary Information:**

The online version contains supplementary material available at 10.1007/s11357-022-00548-w.

## Introduction

Mitochondria are dynamic organelles producing most of the energy required for cellular metabolism and are often referred to as “the powerhouse of the cell.” Loss of mitochondrial function is crucial in the aging and disease processes. Lysophosphatidylcholines (LPCs), a major class of phospholipids, can have positive and negative effects on mitochondria under specific conditions. The effect of LPCs on mitochondria is complex and not fully understood (for review, see [8]. For instance, LPCs can promote the generation of reactive oxygen species (ROS) in mitochondria, which lead to increased oxidative stress and inflammation, especially in older age [7, 15, 17, 22, 30, 34] (for review, see [21]. LPCs also have anti-inflammatory properties [14, 15, 18, 20] (for review see [19]. For example, LPCs 16:0 and 18:1 can activate peroxisome proliferator–activated receptor δ which is involved in mitochondrial metabolism regulation and protects against fatty acid–induced inflammation in human skeletal muscle [18]. On the other hand, LPCs are important for the synthesis of cardiolipin, a diphosphatidylglycerol lipid unique to mitochondria and essential for mitochondrial function. Specifically, the breakdown products of LPCs, such as lysophosphatidic acid (LPA) and phosphatidic acid (PA), are key intermediates in the synthesis of cardiolipin [31]. Cardiolipin is a phospholipid specifically localized in the inner mitochondrial membrane, essential for mitochondrial bioenergetics and signaling pathways. Cardiolipin can shape the curvature of the mitochondrial cristae, affect the assembly and correct functioning of the electron transport chain complexes, and consequently alter ATP and ROS production by mitochondria [9, 28].

Recent data suggest that LPCs measured in the blood may be considered a biomarker of mitochondrial function in skeletal muscle. Lower concentrations of selected long-chain LPCs (i.e., chains composed of at least 12 carbons) are cross-sectionally associated with lower skeletal muscle mitochondrial function [32]. Whether a change in plasma levels of LPCs over time is associated with a parallel change in skeletal muscle mitochondrial function has not been investigated. Finding a longitudinal association may further support the hypothesis that LPCs blood levels are causally related to loss of mitochondrial function with aging, and suggest that LPCs may be targeted for intervention aimed at improving mitochondrial function and, in turn, related aging phenotypes. This hypothesis is consistent with previous studies suggesting that lower plasma concentration of selected LPCs, such as 17:0, 18:1, and 18:2, are also associated with aging phenotypes including slower gait speed and greater mobility decline in middle-aged and older adults [11, 27]. In this study, we aimed to examine longitudinal associations between blood LPCs and skeletal muscle mitochondrial function using data from the Baltimore Longitudinal Study of Aging. We hypothesized that baseline concentrations and changes of LPCs over time would be associated with changes in skeletal muscle mitochondrial function assessed in vivo by ^31^P-magnetic resonance spectroscopy (^31^P-MRS).

## Methods

### Study population

Participants were drawn from the Baltimore Longitudinal Study of Aging (BLSA), a longitudinal study with a continuous enrollment since 1958 [10, 33]. In brief, participants must be free of major chronic conditions and cognitive impairment at the time of enrollment. Follow-up visits occur at different intervals depending on a participant’s age (every 4 years for those younger than 60 years, every 2 years for those aged 60–79, and every year for those aged 80 or older). The National Institutes of Health Institutional Review Board approved the BLSA protocol. All participants provided written informed consent at each BLSA visit.

In this study, data were collected between August 2013 and September 2019. The first concurrent assessment of plasma LPCs and mitochondrial function was considered “baseline” in this analysis. We identified and analyzed 184 adult participants who had two or more measures of plasma LPCs and skeletal muscle mitochondrial function over a mean of 2.4 (SD = 0.9) years. The average number of visits was 2.3 (SD = 0.6) per participant.

### Skeletal muscle oxidative capacity determined by ^31^P-MRS

In vivo ^31^P-MRS measurements of phosphorus-containing metabolites were obtained from the quadriceps muscles using a 3 T Achieva MR scanner (Philips, Best, The Netherlands), as described in detail previously [4, 41].

Participants were positioned supine in the bore of the scanner with a foam wedge underneath the knee to maintain slight flexion (30°), with thighs and hips secured with straps to reduce displacement during exercise. As instructed, participants performed a fast, intense, ballistic knee extension exercise designed to deplete PCr in the quadriceps muscles with minimal acidification, permitting assessment of maximal oxidative phosphorylation [5].

A series of pulse-acquire ^31^P-MRS spectra were obtained before, during, and after exercise using a 10-cm ^31^P-tuned, flat surface coil (PulseTeq, Surrey, UK) that was secured over the vastus lateralis muscle of the left thigh. ^31^P nuclei were excited with 90° adiabatic radio frequency (RF) pulses with an inter-pulse delay time TR = 1.5 s, a four-step phase cycle, and four averages, resulting in a temporal resolution of 6 s. A total of 75 spectra were obtained over the 60 s before, 30 s during, and 360 s after exercise; the total duration of MR data acquisition was 7.5 min [4].

Spectra were processed using jMRUI (version 5.2) and fit in the time domain using a nonlinear least-squares algorithm (AMARES) [26, 37]. Maximum muscular oxidative capacity was assessed as the post-exercise PCr recovery rate constant, *k*_PCr_, which was determined by fitting time-dependent changes in PCr peak area using the following mono-exponential function:

$$\mathrm{PCr}\left(t\right)={\mathrm{PCr}}_{0}+\Delta PCr\times \left.(1-{\mathrm{e}}^{-t/\tau }\right),$$

where PCr_0_ was the PCr signal amplitude at the end of the exercise (i.e., the beginning of the recovery), ΔPCr was the decrease in PCr observed from baseline to the end of the exercise, *τ* was the PCr recovery time constant, and *k*_PCr_ was the PCr recovery rate constant determined as 1/τ [4, 41]. To standardize the measure of oxidative function across different participants, the duration of exercise was carefully optimized by consistently requiring a reduction in PCr peak height of at least 33% compared with initial baseline values [25].

### Collection of plasma

Blood was collected from participants at the National Institute on Aging Clinical Research Unit, Medstar Harbor Hospital in Baltimore, MD, following a standardized protocol, as described previously [40]. Participants were not allowed to smoke, exercise, or take medications before the blood samples were collected. Blood samples were drawn from the antecubital vein between 07:00 and 08:00 am after an overnight fast, then stored immediately at 4 °C, centrifuged within 4 h, and immediately aliquoted and frozen at − 80 °C. The collection of EDTA plasma in the BLSA is consistent with guidelines for biomarker studies [36].

### Measurement of plasma metabolites

Plasma metabolites were measured using liquid chromatography with tandem mass spectrometry (LC–MS/MS). Metabolites were extracted and concentrations were measured using the MxP Quant 500 kit (Biocrates Life Sciences AG, Innsbruck, Austria) following the manufacturer’s protocol for a 5500 QTrap (Sciex, Framingham, MA, USA), as described in detail previously [40]. Of 14 LPCs potentially assessed by the MxP Quant 500 kit, 10 LPCs had all concentration values above the limit of detection and were included in this analysis.

### Statistical analysis

Bivariate correlations of participants’ characteristics with *k*_PCr_ at baseline and the last visit were examined using Pearson correlation coefficients for continuous variables and *t*-tests for categorical variables.

We first examined cross-sectional associations at baseline to confirm previous findings using the MxP Quant 180 kit [32]. We then estimated rates of change in *k*_PCr_ and each LPC per participant using simple linear regression. To examine associations of baseline LPCs and rates of change in LPCs with the rate of change in *k*_PCr_, we used multivariable linear regression. Models were adjusted for baseline age, sex, race, height, and extent of baseline PCr depletion. In sensitivity analyses, we repeated the analysis by using relative measures of LPCs which were significant in the main analyses of the absolute concentration values. The relative measure was calculated as a ratio of the individual concentration to the sum of the concentrations from all 10 LPCs.

All analyses were performed using RStudio version 4.0.2 (Boston, MA). Significance was set at two-tailed *p* < 0.05.

## Results

We analyzed 184 participants who had repeated measures of both plasma LPCs and skeletal muscle mitochondrial function, assessed as *k*_PCr_ via ^31^P-MRS, over an average of 2.4 (SD = 0.9) years. The mean baseline age of participants in this sample was 74.5 years old, 57% were women, and 25% were black (Table 1). On average, the skeletal muscle mitochondrial function measure, *k*_PCr_, declined 0.00002 (SD = 0.002) per year in this analytical sample.

**Table 1:** Participants’ characteristics (*n* = 184)

	Baseline			Last visit		
	Mean (SD) or *N* (%)	Range	Correlations with *k*_PCr_, *p*-value	Mean (SD) or *N* (%)	Range	Correlations with *k*_PCr_, *p*-value
Age, years	74.5 (10.5)	37–98	**0.003**	76.9 (10.2)	42–100	** < 0.001**
Women	104 (57)	-	** < 0.001**	104 (57)	-	** < 0.001**
Black	46 (25)	-	** < 0.001**	46 (25)	-	** < 0.001**
Body mass index, kg/m^2^	26.3 (4.0)	17.6–40.3	0.39	26.5 (4.5)	16.7–41.1	0.62
Height, cm	166.8 (8.3)	146.3–189.7	0.17	166.3 (8.6)	147.1–188.0	0.07
Usual gait speed, m/s	1.18 (0.22)	0.59–1.90	** < 0.001**	1.12 (0.22)	0.36–1.81	** < 0.001**
PCr depletion, %	54.6 (11.1)	33.3–81.1	0.31	58.1 (9.1)	33.2–76.0	0.93
LPC C16:0, µM	126 (28.2)	70.5–257	** < 0.001**	119 (24.5)	70.6–229	** < 0.001**
LPC C16:1, µM	3.4 (1.1)	1.2–8.0	** < 0.001**	3.2 (1.1)	1.5–11	**0.02**
LPC C17:0, µM	2.5 (0.7)	0.8–5.2	**0.002**	2.4 (0.63)	1–4.2	0.07
LPC C18:0, µM	39.6 (9.6)	19.7–81.2	**0.002**	37.8 (7.9)	19.9–59.9	**0.004**
LPC C18:1, µM	26.4 (7.5)	10.4–64.3	** < 0.001**	25.6 (7.4)	9.7–58.5	**0.003**
LPC C18:2, µM	43 (13.5)	16.2–97.4	**0.003**	41.7 (13)	12.8–88.4	0.06
LPC C20:3, µM	2.5 (0.9)	0.9–5.4	** < 0.001**	2.5 (0.9)	0.7–5.5	**0.05**
LPC C20:4, µM	9.4 (3.2)	3.8–19.6	0.43	8.9 (2.7)	3.3–20	0.46
LPC C24:0, µM	0.16 (0.05)	0.08–0.36	0.24	0.17 (0.05)	0.07–0.42	0.88
LPC C28:1, µM	0.41 (0.16)	0.12–1.41	0.45	0.45 (0.16)	0.15–1.04	0.07

Bold number reflects significant correlations at two-tailed *p* < 0.05

We first confirmed the cross-sectional associations published previously except for LPC 18:0 (Semba, et al. 2018). In our study, lower concentrations of several plasma LPCs (16:0, 16:1, 17:0, 18:0, 18:1, 18:2, and 20:3) were cross-sectionally associated with lower *k*_PCr_ at baseline with and without covariate adjustment (Supplementary Table 1). Associations of *k*_PCr_ with LPC 20:4, 24:0, and 28:1 were not significant at baseline (Supplementary Table 1).

**Fig. 1:**
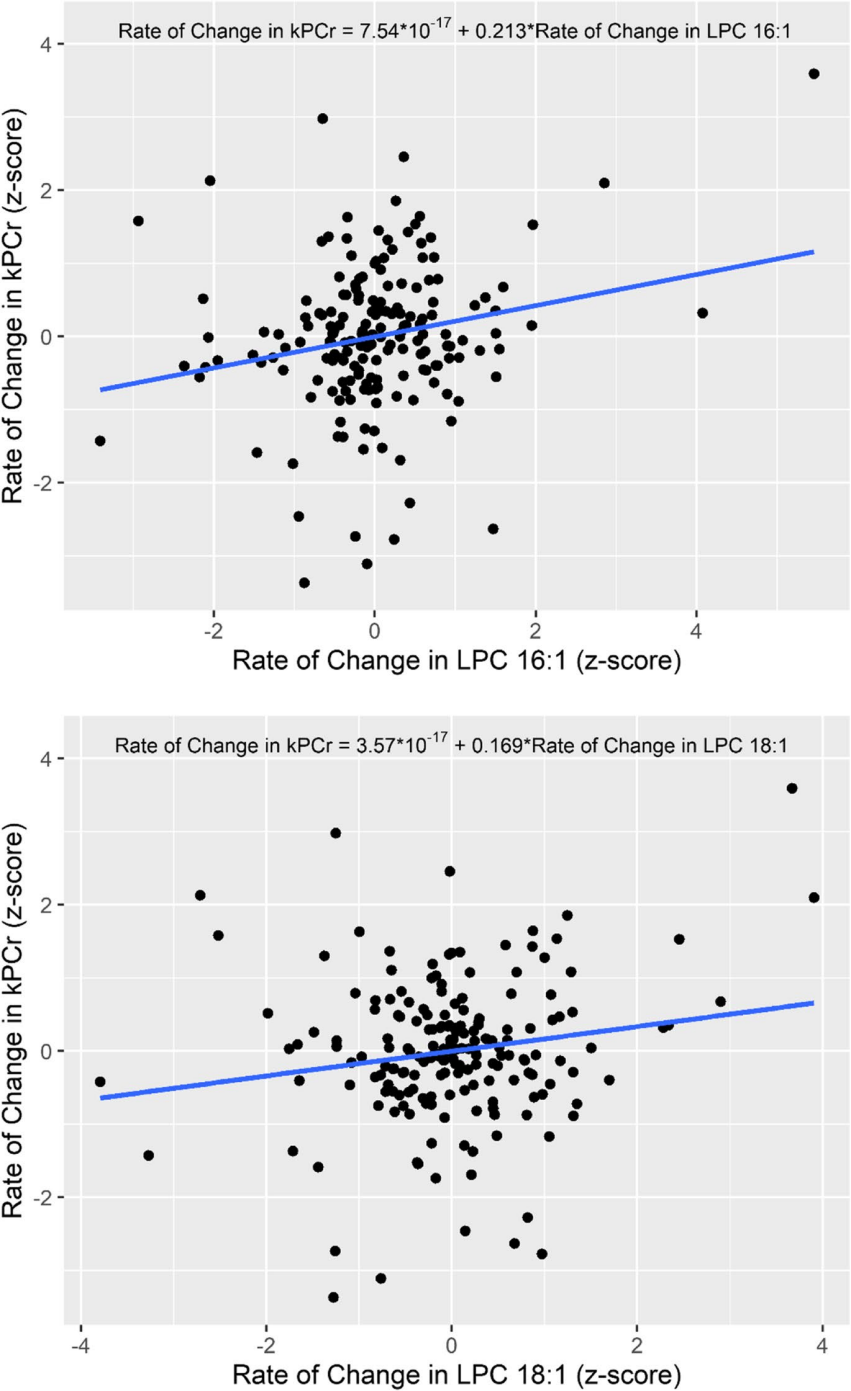
Scatter plots of rates of change in LPC 16:1 and 18:1 with rate of change in *k*_PCr_

In multivariable linear regression models, lower baseline concentration of baseline LPC 16:1 and faster rates of decline in LPC 16:1 and 18:1 were significantly associated with a faster rate of decline in *k*_PCr_ with and without covariate adjustment (in adjusted models: *B* =  − 0.169, 95% CI: − 0.328, − 0.010, *p* = 0.038; *B* = 0.209, 95% CI: 0.065, 0.352, *p* = 0.005; *B* = 0.156, 95% CI: 0.011, 0.301, *p* = 0.035, respectively; Table 2, Fig. 1). Because the lipid concentrations tend to be variable over time, we performed sensitivity analyses by using relative measures of LPCs, calculated as a ratio of the individual concentration over the sum of all LPCs concentration. Results for LPC 16:1 and 18:1 remained similar using the relative measures; lower baseline LPC 16:1 and faster rates of decline in LPC 16:1 and 18:1 over time were associated with a faster rate of decline in *k*_PCr_ (*B* =  − 0.192, 95% CI: − 0.352, − 0.033, *p* = 0.018; *B* = 0.227, 95% CI: 0.084, 0.370, *p* = 0.002; *B* = 0.17, 95% CI: 0.022, 0.318, *p* = 0.024, respectively).

**Table 2:** Longitudinal associations between rates of change in lysophosphatidylcholines and the rate of change in *k*_PCr_ (*n* = 184)

	Model 1: unadjusted			Model 2: covariates adjusted		
LPC species	*β*	95% CI	*p*-value	*β*	95% CI	*p*-value
LPC C16:0	0.118	− 0.027, 0.263	0.11	0.115	− 0.030, 0.261	0.12
LPC C16:1	0.213	0.070, 0.355	**0.004**	0.209	0.065, 0.352	**0.005**
LPC C17:0	0.103	− 0.042, 0.249	0.16	0.116	− 0.030, 0.262	0.12
LPC C18:0	0.084	− 0.062, 0.230	0.26	0.090	− 0.056, 0.236	0.23
LPC C18:1	0.169	0.024, 0.313	**0.02**	0.156	0.011, 0.301	**0.04**
LPC C18:2	− 0.022	− 0.168, 0.124	0.77	− 0.016	− 0.164, 0.132	0.83
LPC C20:3	0.093	− 0.052, 0.239	0.21	0.107	− 0.040, 0.254	0.15
LPC C20:4	0.103	− 0.043, 0.248	0.17	0.104	− 0.043, 0.252	0.16
LPC C24:0	− 0.034	− 0.181, 0.112	0.64	− 0.030	− 0.178, 0.118	0.69
LPC C28:1	− 0.060	− 0.206, 0.086	0.42	− 0.056	− 0.204, 0.091	0.45

*LPCs*, lysophosphatidylcholines. Rates of change in *k*_PCr_ and LPCs were first computed as slopes using simple linear regression and then converted to standardized z-scores. Covariates included baseline age, sex, race, height, and extent of baseline PCr depletion %. Bold numbers reflect significant associations at two-tailed *p* < 0.05

## Discussion

We previously reported that lower levels of specific plasma LPCs are cross-sectionally associated with lower mitochondrial function. Here, we extend our previous findings by demonstrating that baseline LPCs (16:1) and change in LPCs over time (16:1 and 18:1) are associated with a longitudinal change in skeletal muscle mitochondrial function over time in the same direction.

A novel finding in this longitudinal study is that we found that only selected LPC species, especially 16:1, and 18:1, were associated with the change in mitochondrial function, whereas both this and previous studies found more LPC species were cross-sectionally associated with mitochondrial function (including 16:0, 17:0, 18:0, 18:2, 20:3 in addition to 16:1 and 18:1). Longitudinal findings support the causal hypothesis because they track within-individual change over time and reflect a meaningful temporal sequence that cannot be fully captured by cross-sectional analysis. Associations with LPC C16:1 and 18:1 remained robust in analyses of the relative ratio measures. In addition, lower LPC C16:1 at baseline was also associated with subsequent change in skeletal muscle mitochondrial function. Evidence from animal studies has shown that LPC C16:1 (palmitoleic acid 16:1, n-7) is positively correlated with succinate-fueled state 3 mitochondrial respiration [1] and can enhance mitochondrial fatty acid oxidation, oxygen consumption, and ATP content in white adipocyte [6]. Our findings suggest that interventions that increase plasma levels of specific LPC classes may prevent the decline of mitochondrial function that is often observed with aging and chronic diseases. Dietary LPC 18:1 (oleic acid 18:1) can be efficiently incorporated into cardiolipin [35] and contribute to remodeling of the cardiolipin profile [24]. Some animal studies have shown that vitamin E increases LPC 16:0, which can have anti-inflammatory and anti-ROS properties [3, 14, 18, 20, 23], and that the intake of LPC 18:0 protects against obesity [12]. Recent data have shown that a 30-day medium-chain triglycerides intervention induced ketosis, increased concentrations of LPC 16:0, P-18:1, P-18:0, 20:2, and 22:5, and improved cognitive function in patients with mild to moderate Alzheimer’s disease who are not apolipoprotein E ɛ4 carriers [39]. Importantly, increases in LPC P-18:1 were associated with decreases in cognitive impairment scores after the intervention. These findings are supported by previous intervention showing that the ketogenic agent improved cognitive performance in patients with mild to moderate Alzheimer’s disease [13]. Several studies have shown that ketogenic diets can not only improve cognitive function but also affect other metabolic pathways, such as the increased activity of mitochondrial uncoupling proteins, decreased free radical production, improved ATP production in mitochondria, and improved mitochondrial membrane potential [29].

Data are emerging on the important roles of lipids and lipid metabolism in aging and age-related disease, such as Alzheimer’s disease (for review, see [16, 38]. Human intervention studies have shown that the intake of median chain triglycerides and eicosapentaenoic acid/docosahexaenoic acid (EPA/DHA) supplementation significantly increased certain plasma lysophosphatidylcholine concentrations [2, 39]. Future research is warranted to explore what dietary intake of lipids would change circulating lysophosphatidylcholines and thus affect lipid metabolism.

This study has several strengths. Repeated measures of metabolomics and skeletal muscle mitochondrial function allow us to investigate within-individual change over time. Assessment of in vivo skeletal muscle mitochondrial function using MR spectroscopy is state-of-the-art. Plasma samples were collected from fasting participants. In addition, examining both absolute concentration and relative ratio measures of LPCs confirmed the strength of the longitudinal association, specifically LPC 16:1 and 18:1. This study has some limitations. The study sample consists of well-characterized community-dwelling adults who tend to be healthier, more educated, and have higher socioeconomic status than the general adult population. The sample size is modest. Future studies with a larger sample and more repeated measures over a longer follow-up time are needed to confirm the findings of this study. Ultimately, the hypothesis that dietary intervention or supplementation that can increase the plasma level of LPCs may improve mitochondrial function should be tested in clinical trials.

## Supplementary Information

Below is the link to the electronic supplementary material.

Supplementary file1 (DOCX 27 KB)
