# Supplementary material for: Longitudinal associations between blood lysophosphatidylcholines and skeletal muscle mitochondrial function
Source: GeroScience. 2022 Apr 7;44(4):2213–21. doi: 10.1007/s11357-022-00548-w (PMC9616971; doi:10.1007/s11357-022-00548-w)
Supplement: Supplementary file 1 — Supplementary file1 (DOCX 27 KB) [file 11357_2022_548_MOESM1_ESM.docx]

**Supplementary Table 1. Cross-sectional associations between lysophosphatidylcholines and k_PCr_ at baseline (n=184).**

|  | **Model 1: Unadjusted** | | | **Model 2: Covariates Adjusted** | | |
| --- | --- | --- | --- | --- | --- | --- |
| **LPC Species** | **β** | **95% CI** | **P-value** | **β** | **95% CI** | **P-value** |
| LPC C16:0 | 0.301 | 0.162, 0.440 | **<0.001** | 0.244 | 0.105, 0.382 | **<0.001** |
| LPC C16:1 | 0.260 | 0.119, 0.401 | **<0.001** | 0.239 | 0.091, 0.387 | **0.002** |
| LPC C17:0 | 0.228 | 0.085, 0.370 | **0.002** | 0.205 | 0.062, 0.347 | **0.01** |
| LPC C18:0 | 0.230 | 0.088, 0.373 | **0.002** | 0.204 | 0.066, 0.342 | **0.004** |
| LPC C18:1 | 0.358 | 0.221, 0.494 | **<0.001** | 0.329 | 0.194, 0.465 | **<0.001** |
| LPC C18:2 | 0.217 | 0.075, 0.360 | **0.003** | 0.168 | 0.030, 0.306 | **0.02** |
| LPC C20:3 | 0.293 | 0.153, 0.433 | **<0.001** | 0.241 | 0.103, 0.378 | **<0.001** |
| LPC C20:4 | 0.058 | -0.088, 0.204 | 0.43 | 0.025 | -0.117, 0.168 | 0.73 |
| LPC C24:0 | 0.087 | -0.059, 0.233 | 0.24 | 0.094 | -0.044, 0.231 | 0.18 |
| LPC C28:1 | 0.057 | -0.089, 0.203 | 0.45 | 0.082 | -0.065, 0.228 | 0.27 |

Note. LPCs = Lysophosphatidylcholines. Values of k_PCr_ and LPC concentrations were computed as standardized Z scores. Covariates included age, sex, race, height, and PCr depletion %. Bold numbers reflect significant associations at two-tailed p <0.05.
